# Pulmonary Langerhans Cell Histiocytosis with Lytic Bone Involvement in an Adult Smoker: Regression following Smoking Cessation

**DOI:** 10.1155/2015/201536

**Published:** 2015-02-18

**Authors:** B. Routy, J. Hoang, J. Gruber

**Affiliations:** ^1^Division of Haematology and Oncology, McGill University Health Centre (MUHC), Montreal General Hospital, 1650 Cedar Avenue, Montreal, QC, Canada H3G 1A4; ^2^Department of Medicine, Division of Internal, McGill University Health Centre (MUHC), Montreal General Hospital, 1650 Cedar Avenue, Montreal, QC, Canada H3G 1A4; ^3^Department of Medicine, Division of Respirology, McGill University Health Centre (MUHC), Montreal General Hospital, 1650 Cedar Avenue, Montreal, QC, Canada H3G 1A4

## Abstract

Langerhans cell histiocytosis (LCH) is a rare myeloid neoplasm characterized by the proliferation and dissemination of histiocytes. These in turn may cause symptoms ranging from isolated, infiltrative lesions to severe multisystem disease. Pulmonary Langerhans cell histiocytosis (PLCH) presents as a localized polyclonal proliferation of Langerhans cells in the lungs causing bilateral cysts and fibrosis. In adults, this rare condition is considered a reactive process associated with cigarette smoking. Recently, clonal proliferation has been reported with the presence of BRAF V600E oncogenic mutation in a subset of PLCH patients. Spontaneous resolution was described; however, based on case series, smoking cessation remains the most effective way to achieve complete remission and prevent long term complications related to tobacco. Herein, we report the case of an adult woman with biopsy-proven PLCH presenting with thoracic (T8) vertebral bone destruction. Both the lung and the bone diseases regressed following smoking cessation, representing a rare case of synchronous disseminated PCLH with bone localization. This observation underscores the contribution of cigarette smoking as a systemic trigger of both pulmonary and extrapulmonary bone lesions. A review of similar cases in the literature is also presented.

## 1. Introduction

Among myeloid neoplasms, Langerhans cell histiocytosis (LCH), previously called pulmonary histiocytosis X or eosinophilic granuloma of the lungs, remains a rare condition [1]. Characterized by the proliferation and dissemination of histiocytes, this condition is often seen in children and can be limited to an isolated osteolytic lesion in approximately 55% of cases, while the remainder present with multiorgan failure [2]. The accumulation of abnormal bean-shaped nuclei histiocytes in affected tissues is a common pathological finding in LCH. Infiltration of bone marrow derived histiocytes leads to the formation of granulomas surrounded by eosinophilic infiltration and may affect several organs [1, 2]. LCH is considered to be a myeloid neoplasm that arises from aberrant expression of CD1a on the cell surface and is associated with the recently discovered BRAF V600E ontogenetic mutations [3].

In contrast to systemic LCH manifestation found during childhood, pulmonary Langerhans cell histiocytosis (PLCH) presents with respiratory symptoms and primarily in middle-aged adult cigarette smokers. From a radiological perspective bilateral, symmetrical nodules and cysts up to 1 cm in size are most frequently seen on chest imaging [4–8].

Though LCH involves clonally derived cells; PLCH association with BRAF V600E mutation is much weaker. Indeed, some studies have failed to demonstrate evidence of clonal proliferation in patients with lung lesions [5]. Two groups using genomic technology have demonstrated the presence of BRAF V600E mutation in only 1/3 of patients with PLCH [9, 10]. Based on these recent findings, Vassallo et al. proposed a mechanism by which cells are activated upon exposure to cigarette smoking. Then inflammation is worsened with dysregulated T regulatory cells (Treg) and mediates lung injury by promoting the secretion of inflammatory cytokines that activate lung macrophages, which may lead to lung fibrosis [5]. Clinically this close relationship between cigarette smoking and PLCH was highlighted in teenagers with systemic LCH who developed PLCH after starting smoking [6].

The most important aspect in the treatment of PLC patients remains cessation of smoking, which has been associated with complete and long-lasting remission. Nevertheless, in certain cases cigarette cessation is insufficient and immunosuppressive drugs such as prednisone or methotrexate, with or without chemotherapy, are required to control the disease [16–18].

Herein, we describe the case of a 34-year-old woman with biopsy-confirmed, extrapulmonary PLCH involving the T8 vertebra. The patient subsequently had a significant clinical and radiologic regression of two localizations upon smoking cessation. Based on our English-language literature review, this would represent one of the first reports of simultaneous resolution of extrapulmonary PCLH with bone involvement following cigarette cessation.

## 2. The Case

A 34-year-old woman was transferred from Inukjuak, a first nation community in Northern Quebec, to the Montreal General Hospital in Montreal, Quebec, Canada. Her respiratory symptoms included dry cough and dyspnea II/V on Medical Research Council dyspnea scale. She also complained of severe middle back pain radiating to her left ribs that began two months before admission. She smoked a mean of 20 cigarettes per day for 20 years and denied alcohol or drug abuse. Her past medical history was limited to a remote exposure to tuberculosis that did not required treatment. Prior to onset of symptoms, the patient was not taking any medication but began using acetaminophen in the four weeks before admission.

**Figure 1:**
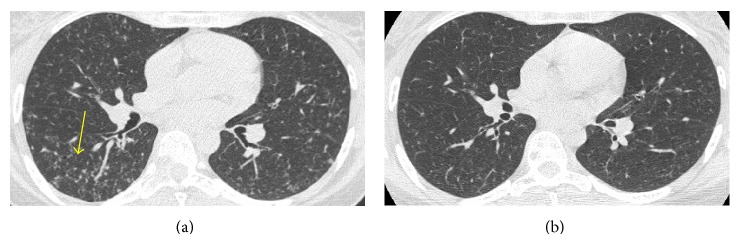
(a) CT of the chest with evidence of bilateral and symmetrical middle lung zone nodules of irregular appearance and cysts. (b) Repeat CT chest 18 months later with disappearance of the nodules and cysts bilaterally.

**Figure 2:**
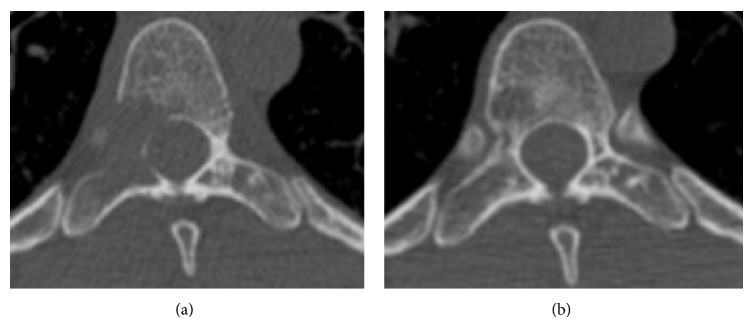
(a) Computed tomography of the spine showed a large solitary osteolytic lesion of thoracic number 8 vertebral body and right pedicle. (b) Repeat CT spine, 18 months later, showed evidence of significance bone regeneration at the T8 osteolytic lesion.

Physical examination was only significant for paraspinal tenderness between T7 and T9 vertebrae. Neurological examination did not reveal motor or sensory dysfunction. Chest auscultation identified normal breath sounds with no adventitious sounds in the absence of cyanosis or clubbing. Laboratory results were within normal values and showed no evidence of hyponatremia, elevated white blood count, or increased LDH. Serum protein electrophoresis and immunofixation were normal. Chest X-ray showed a diffuse bilateral nodular interstitial pattern. A computed tomodensitometry (CT) in contrast to the chest was performed and revealed bilateral reticular-nodular disease in the lungs with the presence of cystic lesions that speared the lung bases (Figure 1(a)). As shown in Figure 1, no lymph node enlargement or pleural effusions were observed. The bone image on the same CT showed a solitary osteolytic lesion at T8 vertebral body and right pedicle, with a two-millimeter margin between the lesion and the spinal cord (Figure 2).

Pulmonary function tests (PFT) demonstrated a restrictive and a marked reduction in diffusing capacity (DLCO 63.5%). A bronchoscopy was performed to identify the cause of the diffuse micronodular lesions in the lungs. Results were negative for bacterial, viral, and Ziehl-Neelsen stains and grocott stains were negative for tuberculosis and pneumocystis jiroveci pneumonia, respectively. A transbronchial biopsy was performed and demonstrated eosinophilic granuloma with the presence of numerous bean-shaped cells with lobulated nuclei. These cells were aggregately stained and were immunohistochemically positive for S100, langerin, and CD1 consistent with the diagnosis of LCH.

To eliminate a neoplastic condition in T8 vertebra, an echo-guided bone biopsy was performed. Pathological findings confirmed the diagnosis of LCH in the body of the vertebra. Based on the absence of both bone collapse and neurological deficits, orthopedic decompression/fixation surgery was not undertaken. Conservative management with smoking cessation and close follow-up was proposed to the patient.

After three months, the dyspnea was reduced to an I/V level on the MRC breathlessness scale, and the diffuse interstitial lesions were no longer present on chest X-ray. The patient reported decreased use of acetaminophen for the back pain, which started to improve four weeks following smoking cessation.

After an 18-month follow-up, the patient experienced a complete resolution of all her symptoms. A repeated CT scan of the chest showed significant decrease of the multiple lung nodules and the bone view revealed an 80% bone regeneration at the site of the initial osteolytic lesion (Figures 1 and 2). Lastly, her lung function completely normalized. Following a final remote interview with the patient four years after this episode, the patient did not resume smoking and remains symptom-free.

## 3. Discussion

This well-documented case illustrates a rare presentation of a synchronous PLCH with extrapulmonary bone involvement. The patient agreed to stop smoking and with this conservative approach only symptoms, PFTs, and radiological imaging improved with concomitant formation of new bone tissue at the site of the large osteolytic lesion on T8 vertebra.

PLCH develops in cigarette smokers due to the thousands of chemicals generated by the combustion of tobacco. This close association was demonstrated in ma murine model. Mice were passively exposed to tobacco smoke and their level of Langerhans cells (LCs) increases by 20-fold [19]. In human reports suggest that bombeside peptides exposed from neuroendocrine lung cells and tobacco glycoprotein, two antigens derived from cigarettes lead to macrophage activation. Subsequently, macrophages secrete tumor necrosis factor alfa (TNF-alfa) and granulocyte-macrophage colony stimulating factor (GM-CSF) which enable LCs expansion locally [5, 20].

In this case report, we hypothesize that the LCs migrated from the lungs to the bone possibly through the thoracic lymphatic tissue. The close vicinity of the pulmonary tissue and the T8 vertebra suggests local lymphatic drainage of reactive cells by locally produced chemokines and/or cytokines such as TNF-alfa and GM-CSF. Subsequently, once the reactive triggers from many cigarette chemicals are discontinued, nonclonal LCs are no longer in an inflammatory microenvironment and may either die by apoptosis or revert to noninflammatory state.

The natural history of PLCH is highly variable and despite tobacco discontinuation, the LCs may remain in an inflammatory status and cells can continue to accumulate. Corticosteroids are the second treatment option of choice according to experts' recommendations, and the paucity of cases does not allow the conducting of clinical trials [16–18]. The benefits of steroids seem to be important especially in the absence of pulmonary fibrosis.

Even in the absence of confirmed clonal proliferation and if the disease is a progressive form of smoking-related interstitial condition, consensuses suggest the use of cytotoxic agents in mono- or combined-drug therapy including vinblastine, cyclophosphamide, cladribine, and etoposide [12, 18]. Lastly, lung transplantation remains an option in patients with severe respiratory failure most frequently secondary to pulmonary hypertension.

We searched the English biomedical literature by using key words: PLCH, multisystemic PLCH, and pulmonary histiocytosis X, in the electronic database systems: PubMed and Medline. Five reports on patients with PLCH presenting with extrapulmonary manifestation were identified and are summarized in Table 1 [11–15].

In all five cases, the PLCH diagnosis was confirmed on histopathology with lung tissue samples. Extrapulmonary lesions were found involving a variety of organs, with the most frequent being bone, skin, and the pituitary gland. Of note, the pathological confirmation of extrapulmonary sites was performed in three cases and assumed based on the radiological findings suggestive of disseminated LCs in two cases.

All investigators encouraged their patients to discontinue cigarette smoking. However, in the majority of cases, further treatments including prednisone or chemotherapies were started preemptively to prevent disease progression in extrapulmonary sites. The majority of patients achieved at least a partial response and remained symptom-free except the patients with pituitary involvement. In two patients with clinical evidence of panhypopituitarism and despite evidence of decreased LCs activity in the lung post treatment, pituitary injury was irreversible and the patients remained on lifelong hormonal replacement.

PLCH remains a challenging disease to treat but the field of histiocytic disorders is evolving quickly. The recent evidence of BRAF oncogenic mutation may offer new therapeutic options with vemurafenib and anti-BRAF targeted therapy approved for the treatment of metastatic melanoma with V600 mutation [21]. By the simultaneous resolution of lesions in lungs and bone, this case report reinforces the strong interaction between the etiology of this disease and cigarette smoking.

## Figures and Tables

**Table 1:** Studies on pulmonary Langerhans cell histiocytosis with extrapulmonary manifestations.

Authors	Number of pts.	Pathological confirmation of PLCH	Extrapulmonary sites	Treatment	Outcome
Vassallo et al. [5, 11]	17	Surgical or transbronchoscopic lung biopsy	Pituitary = 9, bone = 7, skin = 4, lymph node or liver = 4	Prednisone + chemotherapy (vinblastine 7 pts., methotrexate, cyclophosphamide, etoposide, and cladribine 2 pts., respectively	Mixed results with improvement and refractory cases-overall survival shorter than for aged-matched healthy individuals

Karpathiou et al. [12]	1	Thoracoscopic biopsy	Bone: right humerus-biopsy revealed LCH	Smoking cessation	Resolution of the bone without intervention and once patient stopped smoking pulmonary manifestation resolved

Shih et al. [13]	1	Lung biopsy	Bone: skull and one rib discovered on imaging using Technetium-99m	Not reported	Unknown

Nakamura et al. [14]	1	Open lung biopsy	Pituitary: panhypopituitary confirmed on endocrinology testing	Smoking cessation and subsequently methylprednisolone pulse therapy	Disappearance of the pulmonary findings, but patient remained with panhypopituitary dysfunction

Medoff et al. [15]	1	Video assisted surgical biopsy	Bone: left scapular spine and left femur	Smoking cessation + corticosteroid injections in the bone lesions	Almost complete resolution on CT chest 3 months later; bone X-ray 9 months later showed healing with callus formation
